# Endogenous Two-Photon Excited Fluorescence Imaging Characterizes Neuron and Astrocyte Metabolic Responses to Manganese Toxicity

**DOI:** 10.1038/s41598-017-01015-9

**Published:** 2017-04-21

**Authors:** Emily Stuntz, Yusi Gong, Disha Sood, Volha Liaudanskaya, Dimitra Pouli, Kyle P. Quinn, Carlo Alonzo, Zhiyi Liu, David L. Kaplan, Irene Georgakoudi

**Affiliations:** 1 Tufts University, Department of Biomedical Engineering, Medford, MA 02155 USA; 2 University of Arkansas, Department of Biomedical Engineering, Fayetteville, AR 72701 USA

## Abstract

As neurodegenerative conditions are increasingly linked to mitochondrial dysfunction, methods for studying brain cell metabolism at high spatial resolution are needed to elucidate neurodegeneration mechanisms. Two-photon excited fluorescence (TPEF) imaging is a non-destructive, high-resolution technique for studying cell metabolism via endogenous fluorescence of reduced nicotinamide adenine dinucleotide (phosphate) (NAD(P)H) and flavin adenine dinucleotide (FAD). We employed TPEF to study the metabolism of primary rat astrocyte and neuronal cultures under normal growth conditions and in response to manganese (Mn) treatment. Histograms of pixel-wise optical redox ratio, defined as FAD/(FAD + NAD(P)H), revealed three distinct redox distributions and significant differences in their relative weights between astrocytes and neurons. When treated with Mn, both cell types exhibited redox ratio shifts consistent with increased oxidative stress. However, the manner in which the redox distributions was affected was distinct for the two cell types. Furthermore, NAD(P)H fluorescence lifetime imaging revealed an increase in bound NAD(P)H fraction upon Mn treatment for neurons, consistent with enhanced apoptosis. Astrocytes showed a decrease in bound fraction, possibly due to a shift towards glycolytic metabolism in response to impaired respiration. These results exhibit TPEF’s utility for characterizing detailed metabolic changes of different brain cell types in response to neurotoxins.

## Introduction

The brain is an energetically demanding organ; in spite of only making up 2% of the body’s mass, it is responsible for 20% of its energy usage^1^. Proper glucose metabolism is responsible for both maintaining baseline brain electrophysiology and enabling spontaneous brain activity^2^. Different brain cell types have distinct metabolic profiles allowing them to play complementary functions in supporting brain metabolism^3^. Notably, studies from primary cultures of neurons and astrocytes have asserted that neurons primarily generate energy through mitochondrial processes including the tri-carboxylic acid (TCA) cycle and oxidative phosphorylation, while astrocytes also produce significant levels of ATP through glycolysis^3–6^. These distinctions are at least partially attributed to cell types’ different expression levels of genes essential to each energy production pathway^3, 5–7^. These differences enable the cell types to participate in metabolic coupling *in vivo* to support the energy demands of neuronal electrical signaling^8^.

Malfunctions in brain cell metabolism are thought to be in part responsible for debilitating neurodegenerative conditions^9^. Studies are increasingly connecting neurodegeneration to mitochondrial dysfunction; however, exact mechanisms are not yet well understood^9^. Manganism is a neurodegenerative condition caused by environmental exposure to toxic levels of manganese (Mn), which mimics the effects of Parkinson’s Disease (PD)^10^. Studies have reported that Mn poisoning results in depolarization of the mitochondria, inhibition of the respiratory chain, and increases in mitochondrial calcium concentration^11–15^. These alterations may increase reactive oxygen species (ROS) production, which can cause further mitochondrial damage via oxidation^12^. These effects may also accelerate apoptosis^12, 15–19^. Mn affects neurons and astrocytes differently. Both cell types experience inhibition of respiratory activity, but neurons are especially vulnerable to the toxic effects of Mn because they are unable to upregulate other energy production pathways^20^. Astrocytes, however, can alter their metabolism by increasing glycolysis, and in some cases oxidative metabolism, to respond to respiratory chain inhibition and mitochondrial depolarization induced by Mn or other neurotoxins^4, 11, 18, 20, 21^.

Given that different cell types play complementary roles in healthy brain metabolism^3, 8^ and respond differently to neurotoxins^4, 11, 20, 22^, an improved understanding of cell-specific neurodegeneration mechanisms could provide new approaches for diagnosing and treating neurodegenerative diseases. By developing sensitive methods to detect neurodegeneration-related metabolic derangements (for example, oxidative stress^9^) at the cell level, appropriate treatments may be devised and easily screened *in vitro*. Quantitative optical metabolic imaging utilizing two-photon excited fluorescence (TPEF) microscopy provides a non-destructive, high-resolution approach to monitoring cellular metabolic function of cells and tissues via endogenous fluorescence of mitochondrial coenzymes flavin adenine dinucleotide (FAD) and reduced nicotinamide adenine dinucleotide (phosphate) (NAD(P)H)^23^. By selectively exciting the two fluorophores, it is possible to describe relative fluorescence intensities of the NAD(P)H and FAD via an optical redox ratio defined as (FAD)/(FAD + NAD(P)H)^23^. When calculated this way, optical redox ratio values range between 0 to 1 by definition, and have been shown to correlate with liquid chromatography/mass spectrometry (LC-MS) measurements of (NAD^+^)/(NADH + NAD^+^) and/or (FAD)/(FAD + NADH) in epithelial tissue and stem cells^24, 25^. Relative intensity of NAD(P)H and FAD autofluorescence in primary neurons also shows trends consistent with high-performance liquid chromatography (HPLC) measurements of the relative concentrations of NADH and NAD+ in these cells^26^.

While captured NAD(P)H autofluorescence may contain contributions from both NADH and NADPH due to their similar fluorescence properties, the documented associations between optical redox ratio trends and biochemical measurements relating concentrations of NADH to FAD and/or NAD^+^ suggest that fluorescence contributions from NADPH, which are likely small relative to the contributions of bound NADH in the mitochondria^25, 27^, do not prevent optical redox ratio measurements from serving as useful indicators of mitochondrial oxidation status in multiple cell types^24–26^. Optical redox ratios have been used in a variety of applications to describe cellular metabolic activity^23, 25, 28, 29^. In healthy cells, higher optical redox ratio, indicating greater FAD relative to NAD(P)H and likely greater FAD relative to NADH, is indicative of highly oxidative metabolic activity^23^. A lower optical redox ratio, resulting from an increase in NAD(P)H relative to FAD and likely greater NADH relative to FAD, is indicative of enhanced glycolytic activity^23^. In cells subject to stress conditions, redox ratio changes may be associated with other metabolic modulations. For example, multiple spectroscopic and imaging studies have reported increasing redox ratio in relation to apoptosis^28, 30^. Other studies have tied an increase in the ratio of FAD to NAD(P)H fluorescence to oxidative stress^31–33^.

Fluorescence lifetime imaging (FLIM) of NAD(P)H fluorescence can yield further metabolic information about cells. NAD(P)H has been demonstrated to have multiple distinct fluorescence lifetimes based upon NADH conformational folding and binding status^34^. NAD(P)H fluorescence lifetimes of ~0.35 and ~0.75 ns have been reported for stretched and folded free NADH, while lifetimes from ~0.6–6 ns have been reported for varying species of bound NADH^34–36^. Typically, an increased concentration of free NADH is associated with glycolytic activity or reducing conditions, while increased bound NADH is associated with oxidative metabolic activity or oxidizing conditions^35, 37^. NAD(P)H fluorescence lifetime decays may be transformed into phasor space, providing a simple graphical approach for identifying fluorophores with distinct decay profiles present in an image, without requiring any exponential fitting^38^. Decays from a single exponential source fall along a “universal circle” within the phasor plot^29, 38^. Decay profiles representing contributions from two fluorophores (e.g., free and enzyme-bound NADH) fall along a linear trajectory within phasor space, intersecting the universal circle at the two points corresponding to the lifetimes of the decay components^29^. For biexponential NAD(P)H decays, position along the trajectory from “bound” to “free” NAD(P)H can yield information about relative rates of metabolic activities^29, 37^. Previous studies have employed phasor FLIM to delineate shifts in NADH binding status during neural progenitor differentiation, to identify long-lifetime lipid autofluorescence signifying oxidative stress in HeLa cells and cardiomyocytes, and to characterize and monitor metabolism in various types of adipocytes^29, 37, 39, 40^.

Optical redox approaches have previously been used for *in vivo* and *in vitro* spectroscopy and microscopy studies of brain function in healthy tissue and disease models^41–47^. FLIM has been employed in recent studies to track endogenous fluorescence changes during the differentiation of neural progenitor cells, as well as effects of neurotoxins on differentiated PC12 cells^37, 48^. However, studies that have utilized TPEF-based optical redox ratios and FLIM to characterize metabolic differences between primary brain cell types and their responses to Mn toxicity have not been reported. Thus, the goal of our study was to determine whether TPEF of NAD(P)H and FAD could capture metabolic differences between healthy neurons and astrocytes *in vitro*, as well as their responses to Mn toxicity. We first obtained emission spectra of neuron and astrocyte endogenous fluorescence to verify their correspondence to NAD(P)H and FAD. We next compared optical redox ratios of healthy neuron and astrocyte monocultures to determine whether we could detect metabolic differences between the cell types. We then treated both cell types with Mn in order to determine whether TPEF could provide optical signatures of toxicity, as well as insight into the mechanisms of toxicity. Finally, we performed fluorescence lifetime imaging of Mn-treated neurons and astrocytes to further characterize changes in toxicity. These studies demonstrate the usefulness of quantitative TPEF imaging in characterizing brain cell metabolism and investigating the differential impact of neurotoxins on different brain cell types.

## Results

### Cell emission spectra were consistent with NAD(P)H and FAD fluorescence

**Figure 1:**
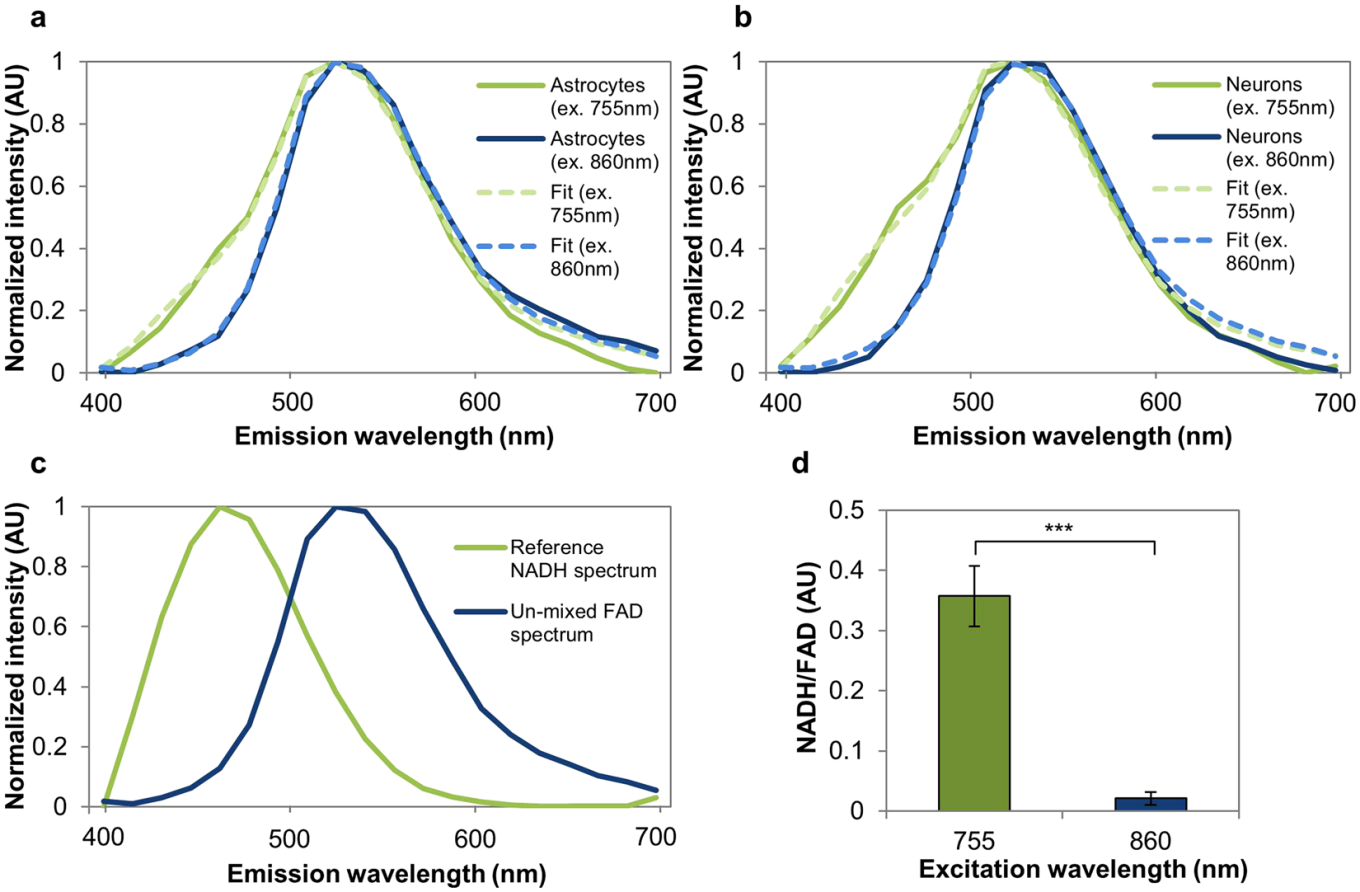
Cell emission spectra show features characteristic of NAD(P)H and FAD. (**a**) Astrocyte and (**b**) neuron emission spectra are broader at 755 nm excitation relative to 860 nm, consistent with emission of both NAD(P)H and FAD at 755 nm, but only FAD at 860 nm. (**c**) Spectral un-mixing with a reference NAD(P)H spectrum yields a second component consistent with FAD’s known emission spectrum. (**d**) The relative weight of NAD(P)H/FAD is significantly higher at 755 nm excitation than 860 nm, consistent with a low excitation efficiency of NAD(P)H at 860 nm. ***p < 0.001.

We collected emission spectra of astrocyte and neuron cultures to assess whether the cells’ autofluorescence could be primarily attributed to NAD(P)H and FAD emission. At 755 nm excitation, we expected both NAD(P)H and FAD to fluoresce^23^. Accordingly, the spectra showed a modulation near 460 nm, consistent with NAD(P)H emission, as well as a peak near 550 nm, consistent with FAD emission^23^ (Fig. 1a,b). At 860 nm, we expected FAD to fluoresce primarily^23^, reflected by the single peak near 550 nm (Fig. 1a,b). The composition of these spectra was further analyzed through spectral unmixing. A previous study from our group reported an NAD(P)H emission spectrum linearly unmixed from emission spectra of human mesenchymal stem cells^49^. We used a spectral unmixing algorithm (non-negative matrix factorization)^50^ on our experimental brain cell spectra with this NAD(P)H spectrum as a fixed component, and obtained a second spectral component consistent with FAD emission (Fig. 1c). After fitting the experimental spectra with these basis components, we compared the fitted relative contributions of NAD(P)H and FAD between excitation wavelengths, and found that the relative contribution of NAD(P)H was significantly higher at 755 nm excitation than at 860 nm, as expected^23^ (Fig. 1d). Based on these emission spectra results, we assumed for the subsequent experiments that images taken at 755 nm excitation and 460 nm emission reflected NAD(P)H fluorescence, and that images taken at 860 nm excitation and 525 nm emission reflected FAD fluorescence.

### Optical redox ratio distributions captured differences in healthy neuron and astrocyte metabolism

**Figure 2:**
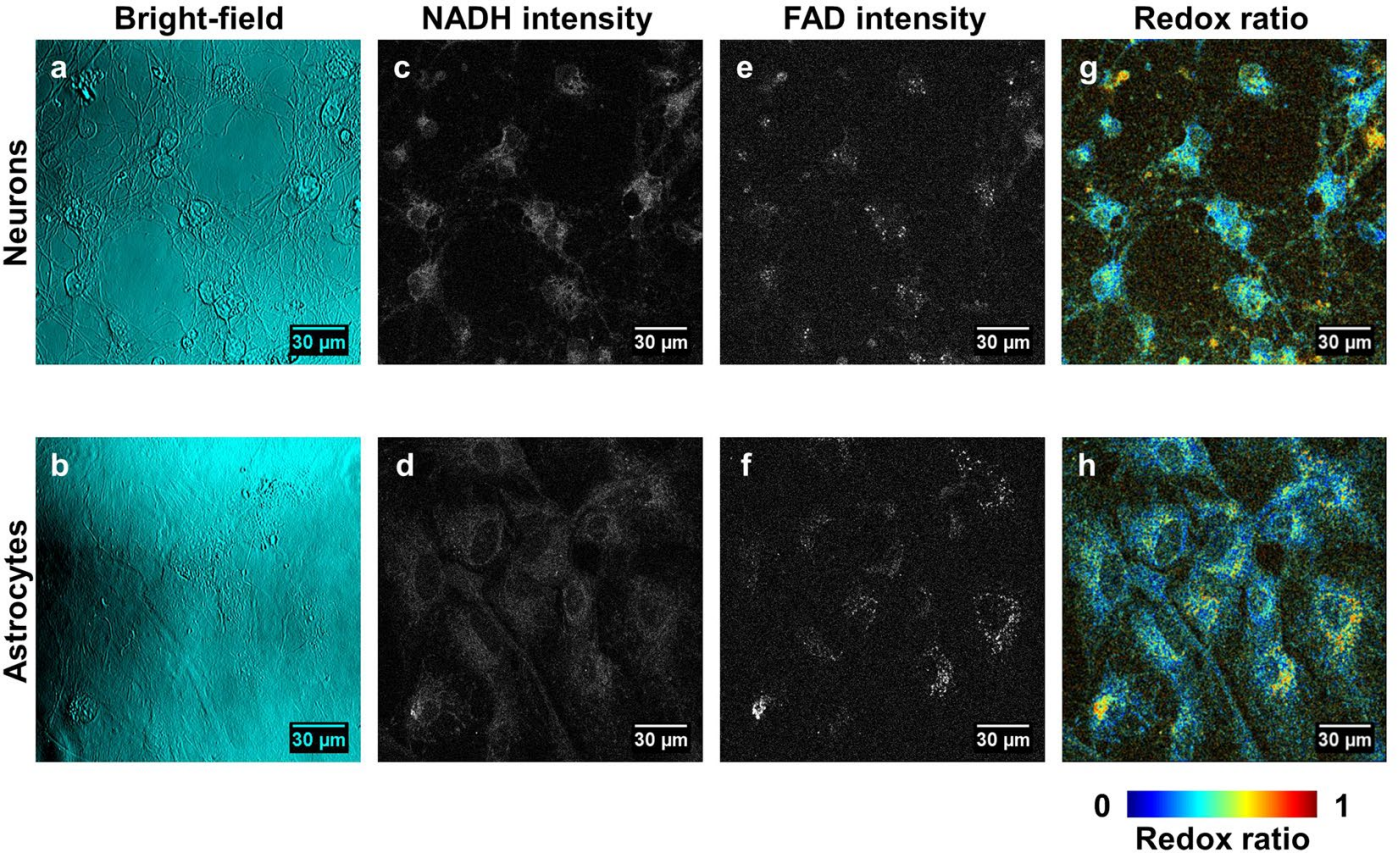
Images of neurons and astrocytes. (**a**) Bright-field images reflect neuron morphology characterized by small cell bodies and highly networked extensions. (**b**) Astrocyte morphology is characterized by a smooth, dense monolayer with spread-out cell bodies. (**c,d**) NAD(P)H fluorescence in neurons (**c**) and astrocytes (**d**) co-localizes with cell bodies. (**e,f**) FAD fluorescence in neurons (**e**) and astrocytes (**f**) localizes closer to the nucleus. (**g,h**) Redox ratio maps of neurons (**g**) and astrocytes (**h**) capture auto-fluorescence trends.

Astrocytes and neurons showed distinct morphologies, endogenous fluorescence patterns, and redox ratio maps (Fig. 2). Neurons were characterized by small cell bodies and highly networked extensions, while astrocytes grew densely with large, spread-out cell bodies (Fig. 2a,b). The dark circular regions within the cells correspond to cell nuclei, as confirmed morphologically through DAPI staining (Supplementary Figures 1 and 2). NAD(P)H fluorescence intensity co-localized with astrocyte and neuron cell bodies, while FAD fluorescence was more intense near cells’ nuclei in astrocytes (Fig. 2c–f). Redox ratio maps further visualized these trends (Fig. 2g,h).

**Figure 3:**
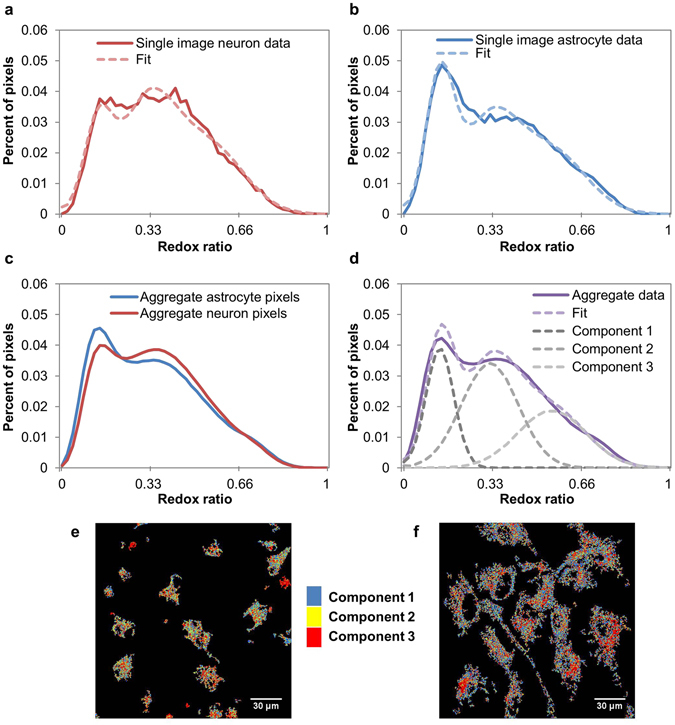
Redox ratio distributions are well-described by the sum of three underlying Gaussian components. (**a,b**) Representative redox ratio distributions for individual neuron (**a**) and astrocyte (**b**) images illustrate how distributions are multimodal and astrocytes have a higher peak at the first mode than neurons. (**c**) Distributions aggregated over all masked images for each cell type further confirm these trends. (**d**) Data from both cell types is combined to determine three underlying Gaussian components. (**e,f**) Images of neurons (**e**) and astrocytes (**f**) are false-colored to show pixels corresponding to each component.

Redox ratio distributions characterized metabolic differences between the cell types. The distribution of redox ratios in representative single images (Fig. 3a,b), as well as aggregated across 250+ images (Fig. 3c) suggest that the distributions were multi-modal. Astrocytes collectively had a greater peak at the first mode, while neurons collectively had a greater peak at the second mode (Fig. 3c). To quantify these differences, pixels were combined across both cell types and the distribution was decomposed into three Gaussian components (Fig. 3d). A three-component model was selected because it yielded a lower Akaike Information Criteria (AIC)^51^ compared with a two-component model, suggesting that the three-component model fit the data better, even when accounting for its increased complexity relative to the two-component model. When cells were false-colored to spatially localize these three components, both cell types contained a combination of the three components, while neurons appeared to have more pixels assigned to component 2 and astrocytes appeared to have more pixels assigned to component 1 (Fig. 3e,f), trends that were confirmed quantitatively in subsequent component weight analysis. Component 1 was centered at the lowest redox ratio value, representing a low ratio of FAD relative to (FAD + NAD(P)H), typically associated with enhanced glycolysis^23^. Components 2 and 3 were centered at medium and high redox ratio values, respectively, representing higher ratios of FAD relative to (FAD + NAD(P)H), typically associated with enhanced oxidative phosphorylation or oxidative conditions^23^.

**Figure 4:**
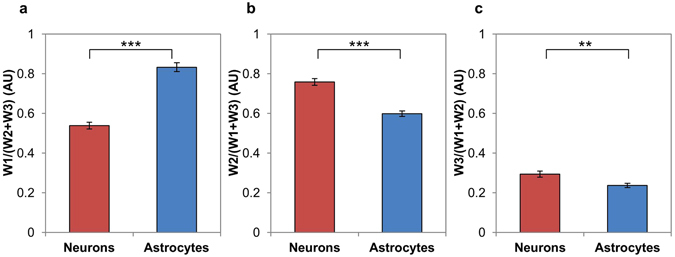
Quantitative comparisons of redox ratio component relative weights. (**a**) Astrocytes have significantly greater relative weight on redox ratio distribution component 1 than neurons. (**b**) Neurons have significantly greater relative weight on redox ratio distribution component 2 than astrocytes. (**c**) Neurons have significantly greater relative weight on redox ratio distribution component 3 than astrocytes. **p < 0.01, ***p < 0.001.

The relative weights of the three components revealed differences between astrocytes and neurons consistent with their known metabolic profiles (Fig. 4a–c). Astrocytes showed a higher relative weight of the low redox ratio component (Fig. 4a), while neurons showed a statistically greater relative weight the medium and high redox ratio components (Fig. 4b,c). Astrocytes’ greater relative weight the low redox ratio component is consistent with previous research suggesting that astrocytes use glycolysis as a more prevalent means of energy production than neurons^3^. Neurons’ greater relative weights on medium and high redox ratio components were consistent with their reliance on oxidative metabolic processes^3^.

### Manganese administration caused morphological and metabolic alterations consistent with apoptosis and oxidative stress

**Figure 5:**
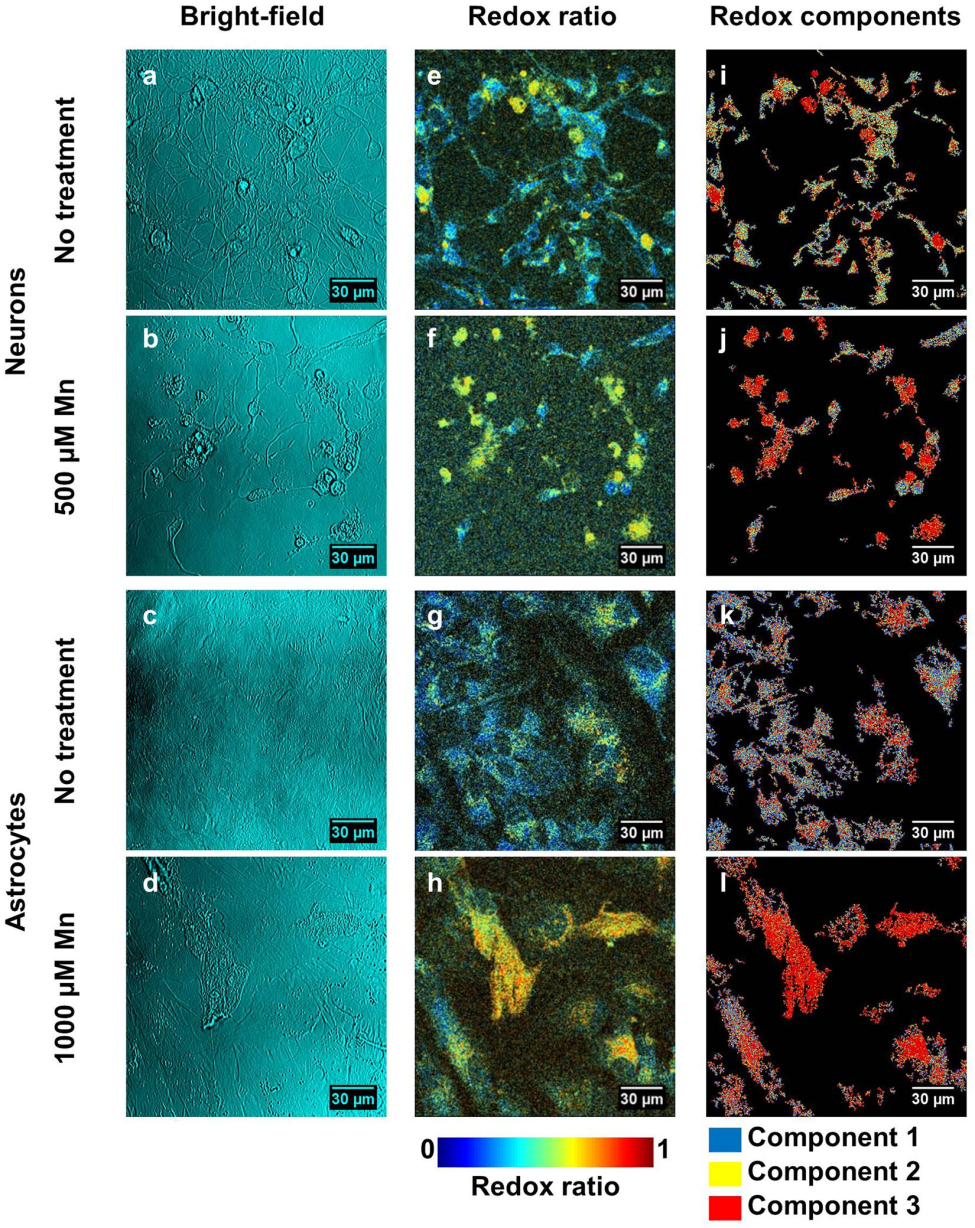
Images revealing cell responses to Mn toxicity. (**a–d**) Bright-field images show morphological changes resulting from Mn toxicity. Neurons treated with Mn (**b**) show axon loss and nuclear blebbing relative to the untreated cells (**a**). Astrocytes treated with Mn (**d**) have a rougher appearance than the untreated cells (**c**). (**e–h**) Redox ratio maps reveal increasing redox ratio with Mn treatment in neurons (**f**) and astrocytes (**h**) relative to controls (**e,g**). (**i–l**) When pixels are colored according to their corresponding redox ratio components, untreated neurons (**i**) and astrocytes (**k**) show a heterogenous mixture of all three components, while treated neurons (**j**) and astrocytes (**l**) are increasingly dominated by component 3.

Culturing neurons and astrocytes with MnCl_2_ resulted in dose-dependent changes to cell morphology and metabolism (Fig. 5, Supplementary Figures 3–4). Consistent with apoptosis, neurons showed nuclear blebbing and axonal fragmentation when treated with 500 μM MnCl_2_ relative to the control (Fig. 5a,b). Astrocytes required treatment with a higher concentration (1000 μM) of MnCl_2_ relative to neurons to yield detectable effects; specifically, treatment with MnCl_2_ resulted in disruption of the smooth, spread out morphology seen in control cultures, yielding a rougher cell appearance (Fig. 5c,d). Redox ratio maps revealed increased redox ratios with MnCl_2_ treatment relative to controls, particularly in cells with morphology that deviated from that of the untreated cells (Fig. 5e–h). Redox component maps further emphasized these alterations; while most untreated cells had a combination of all three redox components (Fig. 5i,k), MnCl_2_-treated cells were frequently dominated by the highest redox ratio component (Fig. 5j,l). While component 3 is observed in control cells (Figs 3e,f and 5i,k), its presence increases substantially in Mn-treated cells (Fig. 5j,l).

**Figure 6:**
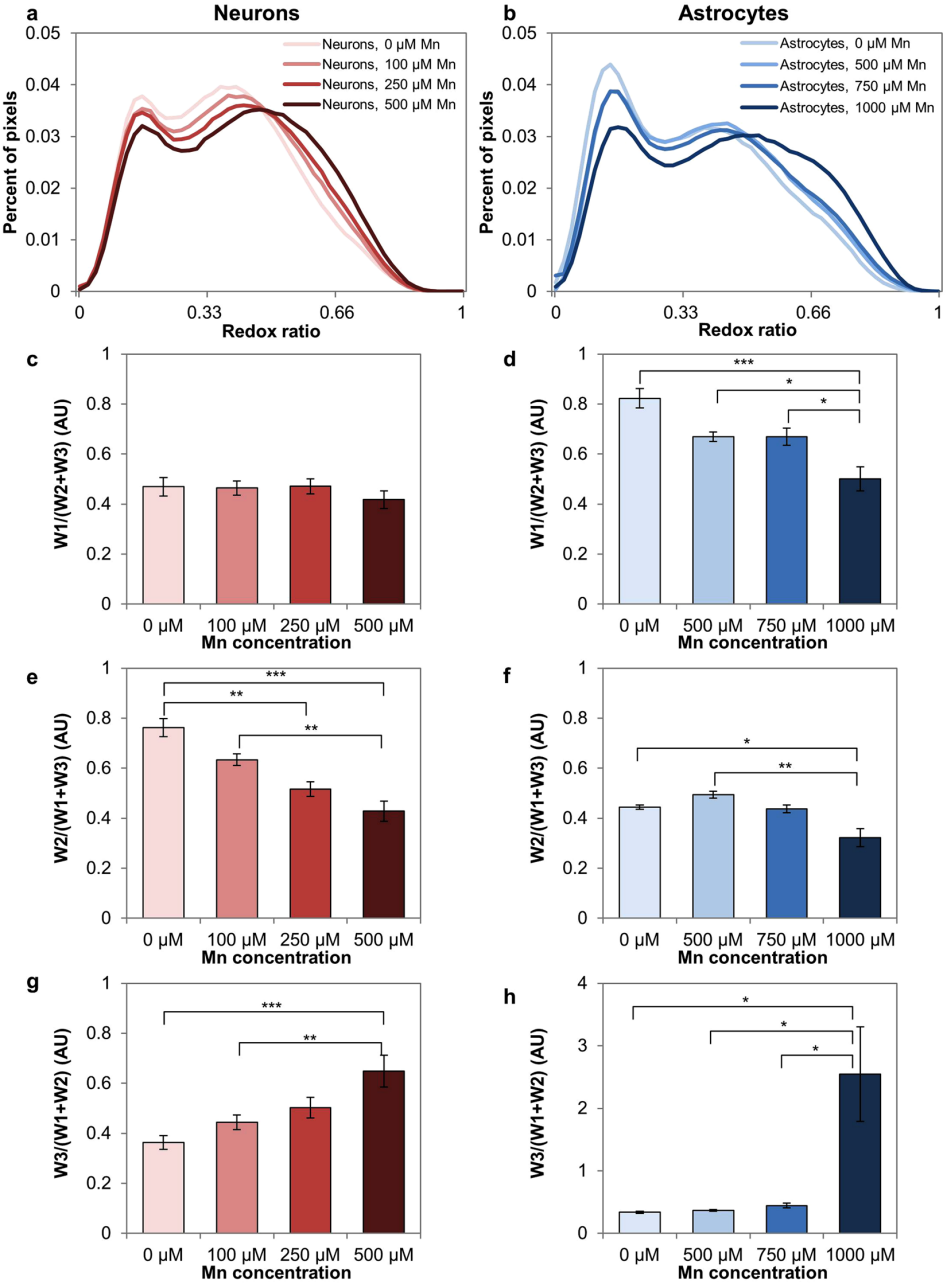
Quantitative comparisons of redox ratio distributions in response to Mn treatment. (**a**,**b**) Redox ratio distributions for neurons (**a**) and astrocytes (**b**) show a broadening in high redox ratio values with increasing Mn dose. (**c**) Relative weight of redox ratio component 1 is consistent in neurons across Mn doses. (**d**) Relative weight of redox ratio component 1 declines with dose in astrocytes. (**e**) Relative weight of redox ratio component 2 declines in neurons across Mn doses. (**f**) Relative weight of redox ratio component 2 declines with dose in astrocytes. (**g**) Relative weight of redox component 3 increases in neurons with dose. (**h**) Relative weight of redox ratio component 3 increases with dose in astrocytes. *p < 0.05, **p < 0.01, ***p < 0.001.

Quantitative analysis revealed increases in the relative weight of the high redox ratio component in both cell types (Fig. 6). When comparing redox ratio distributions of neurons receiving 0, 100, 250, and 500 μM MnCl_2_, the distributions broadened in the higher redox-ratio regions with increasing dose (Fig. 6a). The relative weight of the low redox ratio component stayed consistent with dose (Fig. 6c), while the relative weight of the medium redox ratio component fell with dose (Fig. 6e). The relative weight of the high redox ratio component increased with dose (Fig. 6g).

Astrocytes were treated with 0, 500, 750, and 1000 μM MnCl_2_, and showed a similar dose-dependent broadening in redox distributions at high redox ratio values (Fig. 6b). The relative weight of the low and medium redox ratio components decreased with dose (Fig. 6d,f). Relative weight on the high redox ratio component increased with dose (Fig. 6h).

**Figure 7:**
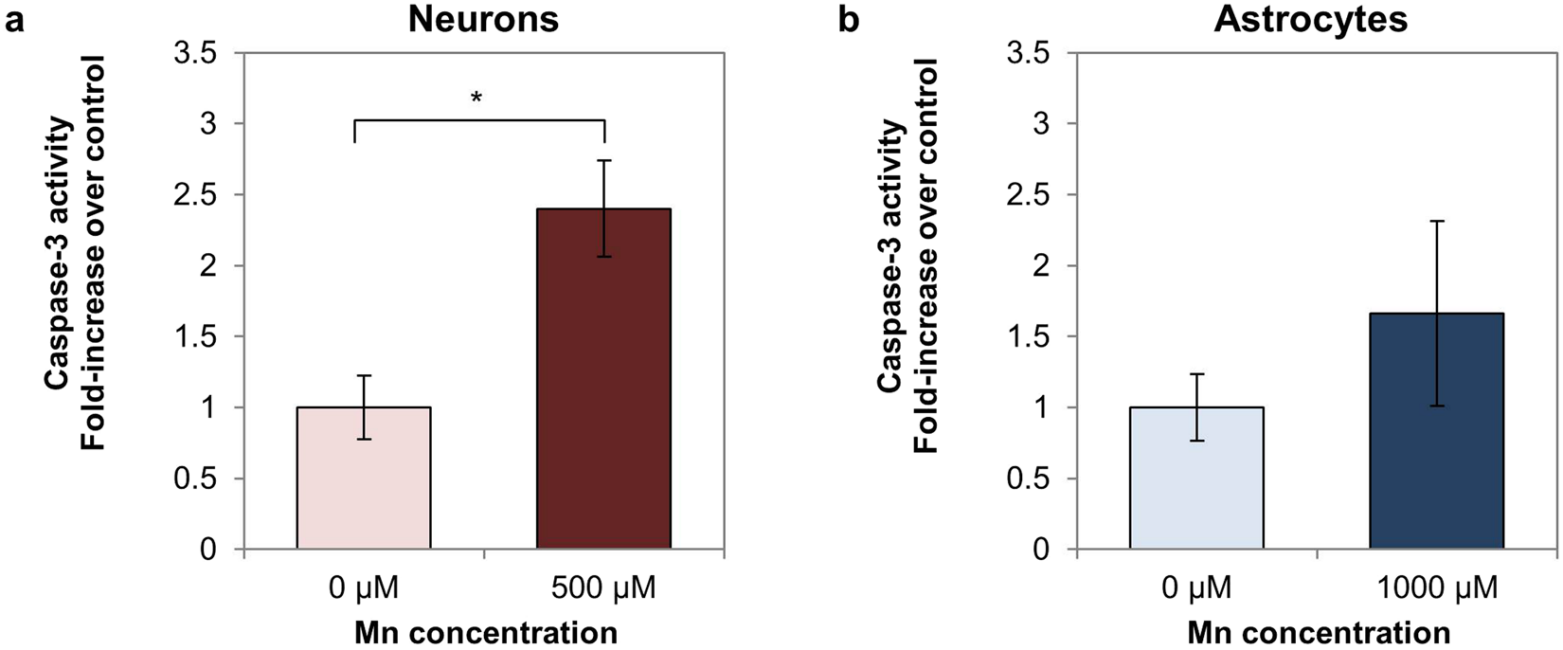
Caspase-3 activity in Mn-treated neurons and astrocytes vs. controls. (**a**) Caspase-3 activity in neurons is significantly elevated in the Mn-treated condition relative to the control. (**b**) Caspase-3 activity in astrocytes is slightly elevated relative to the control, but the difference is not statistically significant. *p < 0.05.

Increased cellular redox ratios have been reported during apoptosis^28, 30^. To assess whether apoptosis might be contributing to the increase in redox component 3 in our results, we performed an assay for caspase-3 in cells treated with MnCl_2_ compared to control cells (Fig. 7). Neurons treated with 500 μM MnCl_2_ had a statistically significant increase in caspase-3 activity (p = 0.0265) relative to control (Fig. 7a). Astrocytes treated with 1000 μM MnCl_2_ showed an increasing trend in caspase-3 activity relative to the control, however the trend was not statistically significant at the p < 0.05 level (p = 0.3680) (Fig. 7b).

### Fluorescence lifetime imaging captured further differences between healthy cells and cells affected by Mn toxicity

**Figure 8:**
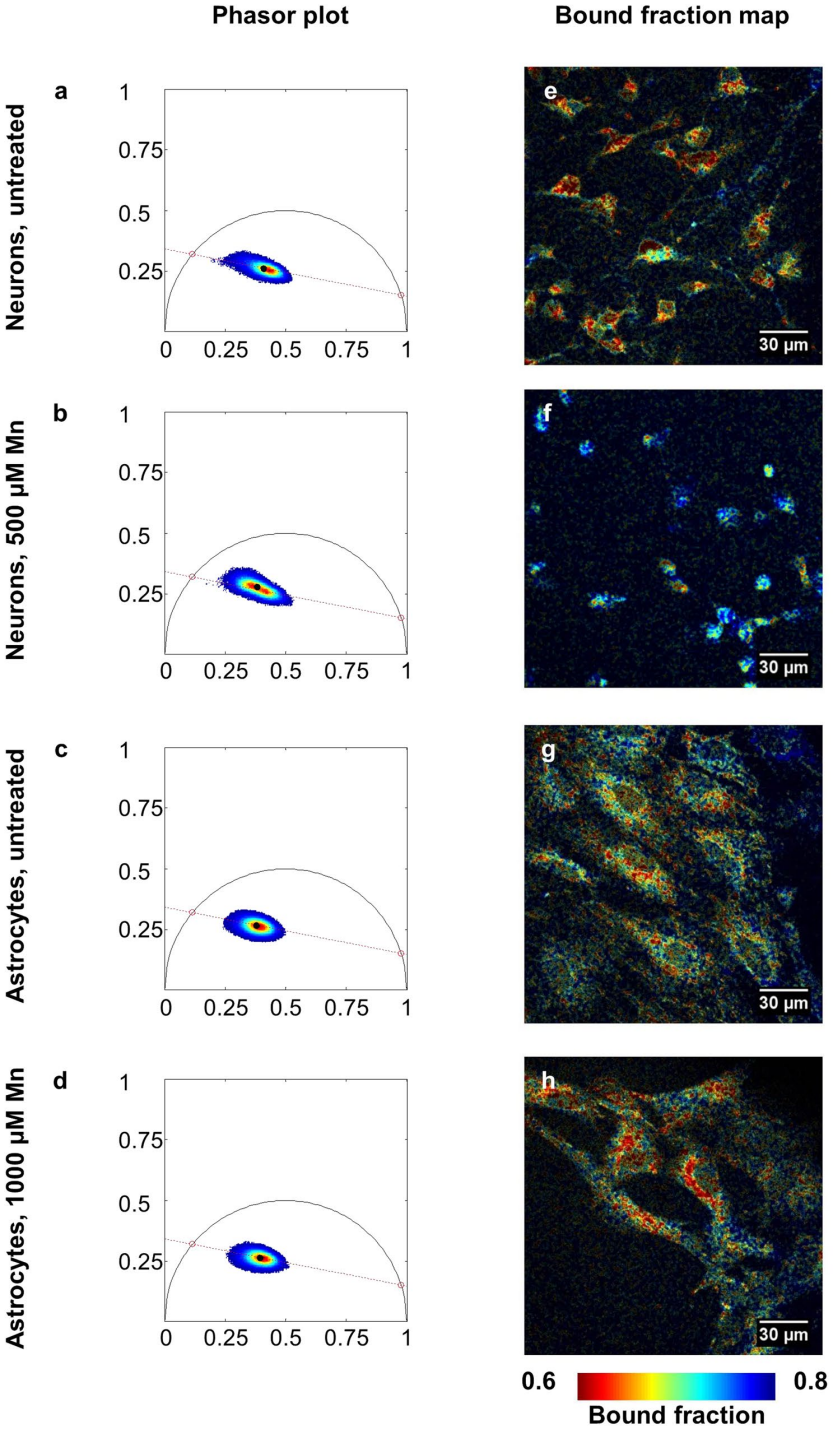
Phasor analysis of NAD(P)H fluorescence lifetime. (**a–d**) Phasor plots of neurons and astrocytes with and without Mn plotted along a standard curve intersecting 5.5 ns and 0.31 ns. (**a**,**b**) Neurons treated with Mn (**b**) have a phasor distribution shifted towards bound NAD(P)H relative to the control (**a**). (**c,d**) Astrocytes treated with Mn (**d**) have a phasor distribution shifted towards free NAD(P)H relative to the control (**c**). (**e–h**) These shifts are echoed in bound fraction maps formed from a representative image from each group.

Neurons and astrocytes were also examined using fluorescence lifetime imaging (FLIM) to further characterize their metabolic differences and responses to MnCl_2_ treatment. Images were analyzed using the phasor approach^38^. Phasors were compiled across all groups to determine a standard curve connecting the short and long lifetime components contributing to the fluorescence signal captured. The curve intersected the universal circle at 0.31 and 5.5 ns (Fig. 8), consistent with ranges reported previously for free and bound NADH, respectively^34–36^. Pixels were compiled across images within groups to show trends in phasor distributions for different cell types and treatments (Fig. 8a–d). These maps revealed that the untreated neuron distribution had a lower mean bound fraction than the astrocyte distribution (Fig. 8a,c). The Mn-treated neuron phasor distribution was shifted towards a higher bound fraction relative to the untreated group (Fig. 8a,b), while the Mn-treated astrocyte phasor distribution was shifted toward the shorter-lifetime end of the trajectory relative to the untreated group (Fig. 8c,d).

**Figure 9:**
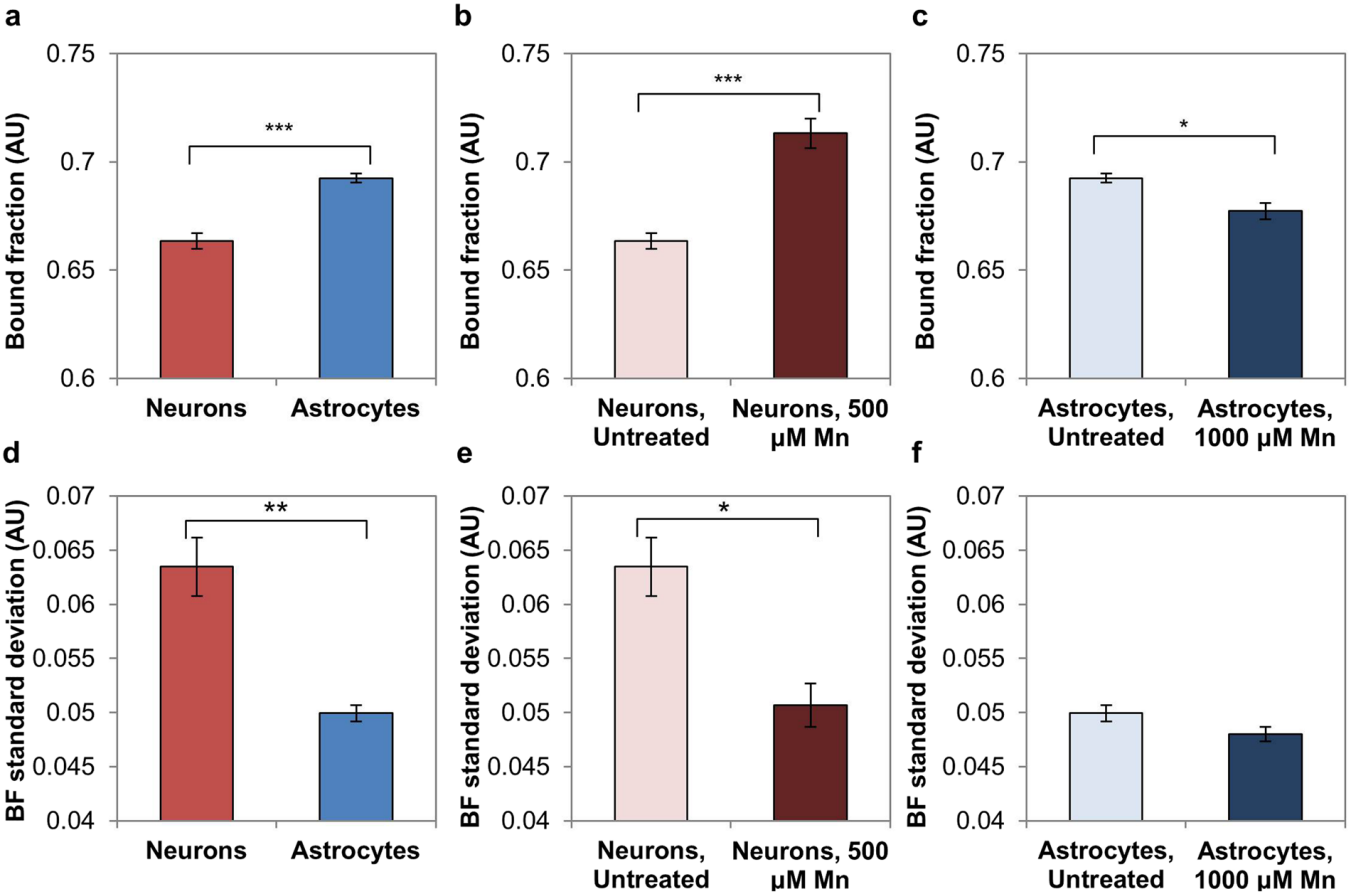
Quantitative comparisons of FLIM metrics between groups. (**a**) Astrocytes exhibit a higher average bound fraction than neurons. (**b**) Neurons treated with Mn have a greater average bound fraction relative to the untreated control. (**c**) Astrocytes treated with Mn have a lower average bound fraction relative to the untreated control. (**d**) Neurons have a greater average image-wise standard deviation of bound fraction than astrocytes. (**e**) Untreated neurons have a greater image-wise standard deviation of bound fraction than neurons treated with Mn. (**f**) Astrocytes have a similar image-wise standard deviation of bound fraction in both conditions. *p < 0.05, **p < 0.01, ***p < 0.001.

False-colored images reflecting the bound NAD(P)H fraction, the relative distance a pixel fell along the trajectory from short to long lifetime, also reflected these shifts. Within neurons the bound fraction increased with Mn treatment, particularly in cells with reduced extensions (Fig. 8e,f). Within astrocytes the bound fraction decreased with Mn treatment, with low bound-fraction values clustering around cell nuclei (Fig. 8g,h). Quantitative comparisons of average bound fraction between groups showed statistically significant shifts (Fig. 9a–c). Astrocytes had greater bound fraction than neurons (Fig. 9a), while neurons had higher image-wise standard deviation of bound fraction than astrocytes (Fig. 9d). Neurons treated with 500 μM MnCl_2_ had an increased bound fraction relative to untreated neurons (Fig. 9b), and decreased image-wise standard deviation of bound fraction (Fig. 9e). Astrocytes treated with 1000 μM MnCl_2_ had a decreased bound fraction relative to untreated astrocytes (Fig. 9c), and lower standard deviation of bound fraction; however, the decrease was not statistically significant (Fig. 9f).

## Discussion

These initial results establish the utility of TPEF imaging of endogenous fluorophores NAD(P)H and FAD for detecting metabolic differences in healthy neurons and astrocytes, as well as their differing responses to Mn toxicity. We demonstrated that pixel-wise optical redox ratios in the two cell types are well-described by the sum of three Gaussian distributions, representing multiple metabolic states and potentially different populations of mitochondria. In healthy neurons and astrocytes, neurons had a significantly greater weight of the medium and high redox ratio components than astrocytes. Astrocytes had a significantly greater weight of the low redox ratio component than neurons. These quantitative metrics reflect that a greater proportion of neuronal pixels fell into medium or high redox ratio distributions than astrocytes, a difference typically associated with increased oxidative vs. glycolytic metabolism^23^. These results are consistent with numerous studies suggesting that neurons tend to utilize oxidative metabolism more exclusively than astrocytes, which also show robust glycolytic activity^3^. Quantitatively analyzing redox ratio histograms is shown to be an effective approach for characterizing metabolic heterogeneity in cell cultures. Astrocytes in particular are known to exhibit a great deal of metabolic heterogeneity, expressing TCA cycle enzymes^52^ in addition to genes up-regulating glycolysis^21^, with metabolic tendencies varying based on spatial location within the cell^53^. Indeed, using a published approach^54^ to calculate the heterogeneity indexes of redox ratio histograms from our neuron and astrocyte images, we quantified the significantly increased metabolic heterogeneity of astrocytes relative to neurons (Supplementary Figure 5). Our approach of analyzing redox ratio histograms allows for delineation of multiple metabolic subpopulations in cell cultures and ultimately shows subtle differences in population weights consistent with literature, which could be overlooked using a less sensitive analysis approach.

FLIM data from astrocytes and neurons showed that astrocytes had a greater average NAD(P)H bound fraction than neurons. Longer NAD(P)H lifetime (or greater NAD(P)H bound fraction) is typically viewed as evidence of more oxidative activity or oxidizing conditions, while shorter NAD(P)H lifetime (or lower bound fraction) is typically indicative of more glycolytic activity or reducing conditions^37, 55^. While this conventional interpretation of our FLIM results would seem to contradict our redox results, it is important to note that differences in lifetime (and by extension, bound fraction) also reflect differences in NADH binding to a range of different substrates^34, 56^. For example, NADH binding to malate dehydrogenase, a TCA cycle enzyme, has been reported to have a mean lifetime around 1 ns, while NADH binding to more complex structures can yield lifetimes from 4–6 ns^34, 56^. Astrocytes have been reported to have greater complex I activity than neurons; however neurons have more efficient coupling between the electron transport chain and energy production^22, 57^. NADH binding to complex I is known to greatly enhance NAD(P)H fluorescence^27^, and our phasors fall along a trajectory between 0.31 and 5.5 ns, consistent with free NADH and NADH bound to a complex structure, respectively. Therefore, we might speculate that our FLIM results are primarily reflecting complex I activity, rather than overall oxidative metabolism. If so, our results are consistent with previous literature showing higher rates of complex I activity in astrocytes relative to neurons^22^. NADH binding differences between the cell types could be also be attributed to other factors. A previous FLIM study of differentiating neural progenitor cells suggested that differentiated neurons have the greatest NADH bound fraction, followed by glial progenitors, and finally neuronal progenitors^37^. While this study did not include differentiated astrocytes, it suggested that glial progenitors had greater bound fraction than neuronal progenitors due to selective binding of NADH to SIRT1, a transcription factor involved in glial differentiation^37^. It is possible that our astrocytes, which were isolated from embryonic day 18 rats, may also have exhibited increased NADH-SIRT1 binding relative to neurons, resulting in a higher bound fraction value. Irrespective of substrate, increased NADH binding in astrocytes could also contribute to the differences observed between neuron and astrocyte redox ratio distributions. If astrocytes showed increased NADH binding relative to neurons, without commensurate increases in other processes involved in oxidative phosphorylation, NAD(P)H fluorescence intensity could be increased relative to FAD intensity, contributing to astrocytes’ greater weight of the low redox ratio component relative to neurons.

Both cell types exhibited morphological, optical redox ratio, and NAD(P)H fluorescence lifetime changes in response to MnCl_2_ treatment. Neurons exhibited nuclear blebbing, neurite loss, a significant increase in the high redox ratio component, and an increased NAD(P)H bound fraction between the control and 500 μM conditions. The increase in the relative weight of the high redox ratio component could be attributed to an increase in oxidative stress or apoptosis, as both have been tied to increased redox ratios in previous studies^28, 30, 31, 33^. An increased redox ratio could reflect increased concentrations of oxidized species (i.e., FAD, NAD+) relative to reduced species (NADH)^24^, suggesting an increase in the presence of oxidizing agents and oxidative stress in cells^31, 33^. Increased reactive oxygen species, which are highly oxidizing, are known to trigger neuronal apoptosis in neurodegenerative conditions^58^. Our neuronal FLIM results are consistent with previously-reported NAD(P)H lifetime increases in apoptotic cells, possibly due to NADH binding to apoptosis-inducing factor (AIF) or other substrates involved in apoptotic pathways^58, 59^. The presence of apoptosis is further suggested by axonal loss and morphological changes, as well as by the increase in caspase-3 levels detected in Mn-treated neuron cultures.

Astrocytes showed morphological and metabolic alterations at 1000 μM MnCl_2_, a higher dose than neurons. These results are consistent with previous research suggesting that neurons are more vulnerable than astrocytes to manganese poisoning^11^. Astrocytes treated with 1000 μM MnCl_2_ exhibited subtle morphological changes relative to the control, an increase in the high redox ratio component, and a decrease in NAD(P)H bound fraction compared to untreated cells. As with neurons, increases in optical redox ratio may be tied to oxidative stress^31, 33^. However, the insignificant caspase-3 changes and decrease in NAD(P)H bound fraction suggest that metabolic alterations, rather than apoptosis, may be responsible for Mn-induced changes in astrocyte auto-fluorescence. A recent FLIM study showed a similar decrease in mean NAD(P)H fluorescence lifetime in PC12-derived neuronal cells treated with MPP+ (1-methyl-4-phenylpyridinium), a complex I toxin used to model Parkinson’s Disease^37^_._ If our FLIM results are primarily reflecting rates of complex I activity, decreasing bound fraction is consistent with complex I inhibition and a movement towards glycolytic energy production in response to Mn-induced respiratory chain inhibition^11, 48^. Interestingly, the results reported from the neuronal PC12 cells align more closely with our astrocyte results than our neuronal results. While our primary neuronal cells show bound fraction increases and morphological changes consistent with apoptosis, the tumor-derived PC12 cells show decreasing fluorescence lifetime and do not show notable morphological changes in response to the neurotoxin. Relative to primary neurons, the neuronal PC12 cells may, like primary astrocytes, have an increased ability to self-protect against toxicity through glycolytic metabolism.

In summary, these preliminary studies exhibit the robustness of TPEF optical redox imaging and FLIM analysis to detect the metabolic differences between brain cell types and their responses to neurotoxins. Our choice to probe endogenous fluorophores allows us to assess living systems non-invasively and with minimal perturbation to the system’s metabolism. High-resolution imaging allows for associations between morphological and metabolic changes, as well as quantitative analysis of metabolic subpopulations. A key contribution of this study is the characterization of metabolic heterogeneity in healthy and Mn-treated cells through quantitative analysis of redox ratio histograms. It is noteworthy that our analysis identified both increases and decreases in different Gaussian redox ratio distributions in response to Mn-treatment (Fig. 6). This approach allowed for characterization of opposing changes to metabolic subpopulations that would be missed by simply examining average redox ratios, which show a modest increase in redox ratio with Mn treatment (Supplementary Figure 6). While the experiments we report aim to establish baseline optical metabolic features of neuron and astrocyte monocultures, future studies should examine reciprocal metabolic activity between the cell types in co-cultures. Further research may also elucidate the more specific sources of redox distribution and FLIM changes through careful chemical modulation of pathways or correlated biochemical assays. Ultimately, these quantitative optical methods may be used in conjunction with existing techniques to probe mechanisms of neurodegeneration in a range of disease models.

## Methods

### Cell Culture

Neurons and astrocytes were isolated from freshly dissected embryonic day 18 (E18) rat cortices as previously described^60^. The Tufts University Institutional Animal Care and Use Committee (IACUC) approved all animal care, methods, and protocols according to NIH guidelines. Neurons were directly plated on glass-bottomed petri dishes coated with poly-D-lysine (Sigma Aldrich, St. Louis, MO)^60^. Astrocytes were expanded until confluent and cryopreserved using standard procedures^61^; for experiments, astrocytes were thawed from frozen and plated on uncoated glass-bottomed petri dishes. Both cell types were cultured in neurobasal medium supplemented with 2% B-27, 2% fetal bovine serum (FBS), 1% Glutamax, and 1% antibiotic/antimycotic (Thermo Fisher Scientific, Waltham, MA). Neurons were allowed to grow for at least 5 days prior to imaging to allow for neurite growth and network formation. Astrocytes were allowed to grow until at least 50% confluent (typically at least 3 days). Media was changed every 2–3 days, with the final change 24 hours prior to imaging. 20 mM HEPES (Thermo Fisher Scientific, Waltham, MA) was added to the media prior to imaging to buffer against changes in CO_2_ levels.

While the midbrain is, perhaps, the most relevant brain area in Parkinson’s disease and, correspondingly, the globus pallidus shows the greatest increases in Mn concentration in cases of Mn poisoning, many additional brain areas are affected by Mn toxicity^62^. Notably, elevated concentrations of Mn have been reported in the cerebral cortex in cases of Mn toxicity, along with biochemical changes suggestive of neurotoxicity^62^. Additionally, several prior Mn toxicity studies in primary cultures have examined cortical neurons and astrocytes^63–65^. We therefore used cortical cells as an established and relevant initial model for understanding the effects of Mn on brain cell metabolism.

Aqueous stock solutions of MnCl_2_ (Sigma Aldrich, St. Louis, MO) were prepared fresh and sterile-filtered. MnCl_2_ solution was added to cell culture media for final concentrations of 100, 250, 500, 750, or 1000 μM Mn 24 hours prior to imaging. Stock solutions were sufficiently concentrated so that the MnCl_2_ solution comprised ≤5% of the total volume of the culture medium added to cells. The untreated cell culture media was determined to have <0.1 μM Mn based on inductively coupled plasma mass spectroscopy.

The range of Mn concentrations tested in both cell types was consistent with previous toxicology studies in cortical neurons and astrocytes^63–65^. A healthy physiological Mn concentration is thought to be 75–100 μM, with clinical symptoms observed in primates when concentrations exceed these levels^63^. Indeed, Mn concentrations ranging from 100–1000 μM have shown concentration-dependent cell toxicity in previous studies of primary cortical neuron and astrocyte cultures^63, 64^. In our experiments, we initially tested concentrations between 100–500μM in both cell types, but saw no dose-dependent effects in astrocytes (data not shown). Therefore, in order to capture the dose-dependent metabolic impact of Mn on astrocytes, we tested a higher concentration range of 500–1000 μM. The increased concentration of Mn required to produce toxicity in astrocytes is consistent with previous reports that neurons are more vulnerable to Mn toxicity than astrocytes^20^.

Caspase-3 content of Mn-treated cells was determined using a colorimetric assay (R&D Systems; Minneapolis, MN) as described in the manufacturer’s protocol. In brief, cells were grown as previously described and treated with either fresh media or fresh media supplemented with 500 μM (neurons) or 1000 μM (astrocytes) MnCl_2_ 24 hours prior to performing the assay. The cells were lysed and centrifuged, and the supernatant was collected for analysis. The caspase-3 colorimetric substrate and reaction buffer were added to supernatant samples and blank samples. Samples were incubated for 2 hours, and the colorimetric change was quantified using a plate reader by measuring absorbance at 405 nm. The blank sample values were subtracted from the control and Mn-treated sample values to provide corrected absorbance values. N = 3–9 cell lysate samples were evaluated per experimental condition and results were expressed as a fold-increase over the control value.

### Imaging

Emission spectra were taken using a Leica SP2 confocal microscope fitted with a Ti:Sapphire laser (Mai Tai, Spectra-Physics, Santa Clara, CA). Light was focused to the sample using a 63x objective (NA 1.2 water-immersion), and neutral density filters were used to achieve a power of 20–30 mW at the stage. Images were 512 × 512 pixels, representing a 238 × 238 micron field of view. Cells were excited at 755 and 860 nm, and fluorescence images were acquired at emission wavelengths centered from 400–700 nm with a bandwidth of 20 nm in 20 steps using the microscope’s built-in prism spectrophotometer to scan the wavelengths detected by the de-scanned photomultiplier tube (PMT). Multiple spectra were taken for each cell type to verify spectral shape, and n = 3 spectra were averaged to give the final reported spectra for each group.

Redox ratio images were taken with a Leica SP2 confocal microscope fitted with a Ti:Sapphire laser. Laser light was focused to the sample using a 40x objective (NA 1.1 water-immersion), and neutral density filters were used to achieve a power of 20–25 mW at the stage. Samples were excited with light at 755 nm and 860 nm and fluorescence images were formed using non-descanned detectors (NDDs) filtered to collect light at 460 +/− 20 nm (ET460/40M-2P; Chroma, Bellows Falls, VT) and 525 +/− 25 nm (ET525/50M-2P; Chroma, Bellows Falls, VT). Images were 512 × 512 pixels, with the zoom feature of the built-in Leica Control software employed to achieve a 187 × 187 micron field of view. Images were formed based on an average of 12 scans. 3–6 non-overlapping images were taken per petri dish or well, and n = 3 dishes were imaged per cell type and treatment condition in each experimental repetition. Redox results reflect summaries of data from at least two experimental repetitions.

Fluorescence lifetime (FLIM) images were taken using a custom-built two-photon microscope fitted with a Ti:Sapphire laser. Light was focused on the sample using a 40x objective (NA 1.1 water-immersion), and a half-wave plate was adjusted to achieve a power of 20–25 mW at the stage. Samples were excited at 755 nm and the resulting TPEF signal was collected using a PMT with a filter centered at 460 nm +/− 20 nm (Chroma, HQ460/40M-2P; Chroma, Bellows Falls, VT). Time-correlated single photon counting (SPC-150; Becker & Hickl, Berlin, Germany) was employed to obtain time-resolved fluorescence decay information. Images were 512 × 512 pixels, representing a 184 × 184 micron field of view, and were acquired over a 70 s integration time. A calibration image of umbelliferone (7-hydroxycoumarin) was taken for each experimental day and showed the characteristic mean lifetime of 5.1 ns. For the cell samples, 4–6 non-overlapping images were taken per petri dish and n = 3 petri dishes were imaged per cell type and treatment group per experimental repetition. FLIM results reflect summaries of data from at least two experimental repetitions.

### Analysis

#### Spectra

Emission spectra were obtained by first determining the average fluorescence intensity of images acquired at each emission wavelength. Spectra taken at 755 nm excitation were corrected for scattered excitation light by subtracting a scaled spectrum of a scattering sample from the raw spectra. The spectra were then scaled between 0 and 100%. To assess the relative contributions of NAD(P)H and FAD to the emission spectra taken at 755 nm and 860 nm excitation, we used an NAD(P)H reference spectrum^49^ to un-mix our experimental spectra into two components using non-negative matrix factorization^50^. The factorization was performed 100 times per image and the best-fit result was recorded, giving the weights of the two components yielding the best fit to the experimental data. The second, un-mixed component matched the expected spectral shape of FAD, with an emission peak near 525 nm.

#### Redox Ratio Analysis

For redox analysis, images were processed in several steps. For a given field, the 755 nm excitation, 460 nm emission (NAD(P)H channel) and 860 nm excitation, 525 nm emission (FAD channel) were normalized for gain and power, background-subtracted, and spatially co-registered by determining the shift maximizing correlation between the two channels^25^. A cell mask was determined by first applying a Gaussian low-pass filter to the NAD(P)H and FAD channels and then enhancing their contrast by raising their intensity values to the 1.75 power. The MATLAB multithresh function was then used to segment each channel into three intensity levels by the Otsu method, which chooses thresholds minimizing variation within each level. Because the NAD(P)H channel included separate moderate and high-intensity pixel populations within cells, we used the lower of the two multithresh thresholds to form a binarized mask of the NAD(P)H channel. We generated an FAD channel mask by applying the higher of its two intensity thresholds because cell pixels in this channel were more uniformly high-intensity and utilizing the lower of the two thresholds resulted in inclusion of significant background regions. The masks for the two separate channels were then combined through addition to generate an overall cell mask, and the MATLAB bwareaopen function was applied to remove small objects with fewer than 50 connected pixels. The mask also excluded saturated pixels and pixels not attributable to FAD or NAD(P)H fluorescence (e.g., with significant fluorescence in the 860 nm excitation, 460 nm emission channel). Pixel-wise redox ratios were calculated by taking a ratio of the normalized FAD intensity to FAD + NAD(P)H intensity.

Redox ratio histograms were generated by first applying a spatial Gaussian low-pass filter to the redox maps to reduce noise. Masked, filtered redox maps were used to generate histograms with 50 bins with redox ratio values between 0 and 1. The resulting histograms were normalized so that the sum across all bins added up to 100%. In histograms summarizing data from multiple experiments, the individual experiments were given equal weight and the resulting histograms were normalized to sum to 100%.

To determine the basis redox ratio components for the redox histograms, we first compiled all of masked, spatially filtered redox ratio pixels from astrocyte and neuron monoculture images. This data represented >250 images across 9 experiments, each containing n = 3 separate cultures of astrocytes and neurons. We used the gmdistribution.fit function within MATLAB to fit Gaussian mixed models to the aggregate histogram assuming 2, 3, and 4 underlying components. The 2- and 3-component models converged, and the 3-component model yielded a lower AIC as reported by MATLAB^51^ than the 2-component fit, suggesting that the 3-component model provided a better fit than the 2-component model. The 4-component fit did not converge. For this reason, a 3-component Gaussian mixed model was used to estimate the three distributions underlying the aggregate redox histogram. The fitting was performed 10 times, and the mean and standard deviations of the three components from each fit were averaged across the 10 iterations to determine the basis components. Redox component maps (Fig. 5i–l) were generated by assigning each pixel to one of the three components, with cutoffs for the three groups defined by the midpoints between the means of components 1 and 2, and components 2 and 3. We then determined the image-by-image relative weights of these redox basis components. We fit the fixed basis components to individual image histograms and normalized the relative weights of the three basis components to add up to 100%. Image-wise ratios of the component weights (i.e., W1/(W2 + W3), W2/(W1 + W3), and W3/(W1 + W2)) were used to quantify and statistically test changes in the redox histograms between groups.

#### FLIM Analysis

FLIM analysis was performed using custom MATLAB software, which converted the time-resolved fluorescence decays into phasor space using sine/cosine transforms^29^. To account for the instrument response function (IRF), a reference image of 7-hydroxycoumarin was first transformed into phasor space to determine the phasor rotation and modulation necessary to position the reference phasor on the unit circle according to its known lifetime (i.e. 5.1 ns, confirmed with exponential fitting)^29, 66^. We then calculated the IRF-corrected phasor coordinates (g, s) for each pixel in each cell image using 2 × 2 pixel binning. We masked the images based on Otsu thresholding of the image intensity, and aggregated the masked, pixelwise phasors across images to form overall phasor plots for groups. A line was fit to the aggregate phasor plot for all of the data in order to determine the “standard curve,” which had intersections with the universal circle corresponding to ~0.31 and 5.5 ns. To determine individual images’ bound fraction values, their average phasor (g, s) values were calculated and projected onto the standard curve. Bound fraction was defined as the distance between the projected point and the standard curve’s short-lifetime intersection with the circle, divided by the length of the standard curve within the circle^66^. To obtain bound fraction standard deviation values for individual images, we projected each masked pixel’s phasor coordinates (g, s) to the standard curve, calculated pixel-wise bound fractions, and determined the bound fraction standard deviation for pixels with non-zero bound fraction values.

### Statistics

Statistical software (JMP 12) was used to evaluate differences in means of image-wise metrics between groups. First, image-wise values were split by group and experiment to identify and exclude outliers (<12% of data). Outliers were identified as values greater than 1.5 times the interquartile range above the 75^th^ quantile of the data, or 1.5 times the interquartile range below the 25^th^ quantile of the data. Then, a mixed model was specified, including fixed effects (e.g., cell type, Mn treatment), as well as random effects where applicable (e.g., petri dish, experimental day). The model was estimated using a restricted maximum likelihood (REML) method, and an F-test was used to assess the significance of the fixed effects. If an effect was significant, a post-hoc Tukey Honest Significantly Different (HSD) test was applied to determine the significance of differences between groups. In graphs, error bars indicate standard error, and significance is indicated using asterisks *p < 0.05, **p < 0.01, ***p < 0.001.

## Electronic supplementary material

Supplementary Information
