# Supplementary material for: Endogenous Two-Photon Excited Fluorescence Imaging Characterizes Neuron and Astrocyte Metabolic Responses to Manganese Toxicity
Source: Sci Rep. 2017 Apr 21;7:1041. doi: 10.1038/s41598-017-01015-9 (PMC5430620; doi:10.1038/s41598-017-01015-9)
Supplement: Supplementary file 1 — Supplementary Information [file 41598_2017_1015_MOESM1_ESM.pdf]

SUPPLEMENTARY INFORMATION

Endogenous Two-Photon Excited Fluorescence Imaging Characterizes Neuron and Astrocyte Metabolic Responses to Manganese Toxicity

Emily Stuntz[1], Yusi Gong[1], Disha Sood[1], Volha Liaudanskaya[1], Dimitra Pouli[1], Kyle P. Quinn[1,2], Carlo Alonzo[1], Zhiyi Liu[1], David L. Kaplan[1], Irene Georgakoudi[1*]

1. Tufts University, Department of Biomedical Engineering, Medford, MA 02155,USA

2. University of Arkansas, Department of Biomedical Engineering, Fayetteville, AR 72701, USA

* Irene.Georgakoudi@tufts.edu

SUPPLEMENTARY METHODS:

Immunohistochemical Staining of Astrocytes and Neurons:

Astrocyte and neuron monocultures were fixed with 4% paraformaldehyde for 10 minutes and washed with phosphate buffered saline (PBS) three times. A blocking solution consisting of goat serum, triton, bovine serum albumin, and PBS was applied to the cells for one hour at room temperature. Primary antibodies for beta-III tubulin and glial fibrillary acidic protein (GFAP), produced in rabbit and mouse, respectively, were added to the cells overnight under refrigeration at four degrees Celsius. Unbound primary antibodies were washed from the fixed cultures three times with PBS. Secondary goat anti-mouse antibodies (AlexaFluor-488) were added to label the GFAP, and goat anti-rabbit antibodies (AlexaFluor-594) were added to label the beta-III tubulin. Unbound primary antibodies were washed from the cultures three times with PBS. 10 minutes prior to imaging, DAPI (NucBlue Fixed Cell ReadyProbes Reagant) was added to the cells. All supplies were obtained from ThermoFisher (Waltham, MA).

Fluorescence imaging:

Images were taken with an inverted fluorescence microscope (Keyence, BZ-X700 series, Itasca, IL), using a 20x objective (NA 0.45 air-immersion). Images were 1920x1440 pixels, representing a 725x545 micron field of view. DAPI fluorescence was captured by using a filter set (OP-87762, Keyence,

Itaska, IL) optimized for DAPI imaging – filters limited excitation light to 360nm +/- 20 nm, and a dichroic filter passed wavelengths greater than 400nm to the emission filters, which passed wavelengths of 470nm +/- 20 nm to the detector. GFAP fluorescence was captured by using a filter set (OP-87763, Keyence, Itaska, IL) optimized for GFP imaging – filters limited excitation light to 470nm +/- 20 nm, and a dichroic filter passed wavelengths greater than 500nm to the emission filters, which passed wavelengths of 525nm +/- 25 nm to the detector. Beta-III tubulin fluorescence was captured by using a filter set (OP-87765, Keyence, Itaska, IL) optimized for Texas Red – filters limited excitation light to 560nm +/- 20 nm, and a dichroic filter passed wavelengths greater than 595nm to the emission filters, which passed wavelengths of 645nm +/- 40 nm to the detector.

Heterogeneity Analysis:

To compare the redox ratio heterogeneity between images of neurons and astrocytes, we calculated a heterogeneity index from each image's redox histogram using a previously published approach[1]. For each image, we calculated average redox ratio within the masked region, and also fit a 3-component Gaussian mixed model to the image's redox histogram to obtain the means and weights of the three image-wise subpopulations. Heterogeneity index was calculated as $-\sum d_i p_i \ln p_i$, where d_i represents the distance between the overall image mean redox ratio and the mean of subpopulation i, and p_i represents the weight of subpopulation i.[1] Heterogeneity index is a relative metric, and a larger heterogeneity index is indicative of greater heterogeneity.[1] Upon obtaining heterogeneity indexes for each image, we used the same statistical approach described in the main paper methods to compare mean heterogeneity indexes between neurons and astrocytes.

SUPPLEMENTARY FIGURES:

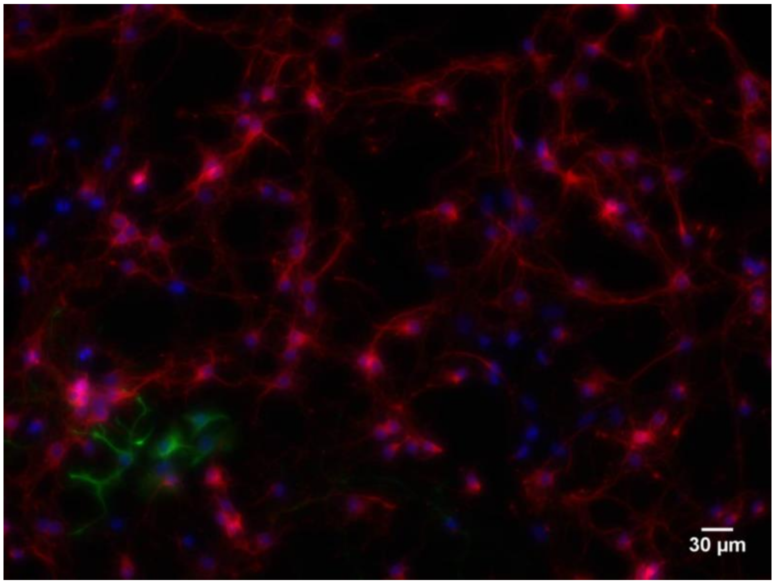

Supplementary Figure 1: Neuronal cultures stained with nuclear marker DAPI (blue) and immunofluorescent markers for structural protein beta-III tubulin (red) show characteristic circular morphology of nuclei within neuron cell bodies. Immunofluorescent marker for GFAP (green) reveals the presence of some astrocytes in the neuronal cultures, likely due to the presence of FBS in the cell culture media. A high level of cultural purity is, however, preserved.

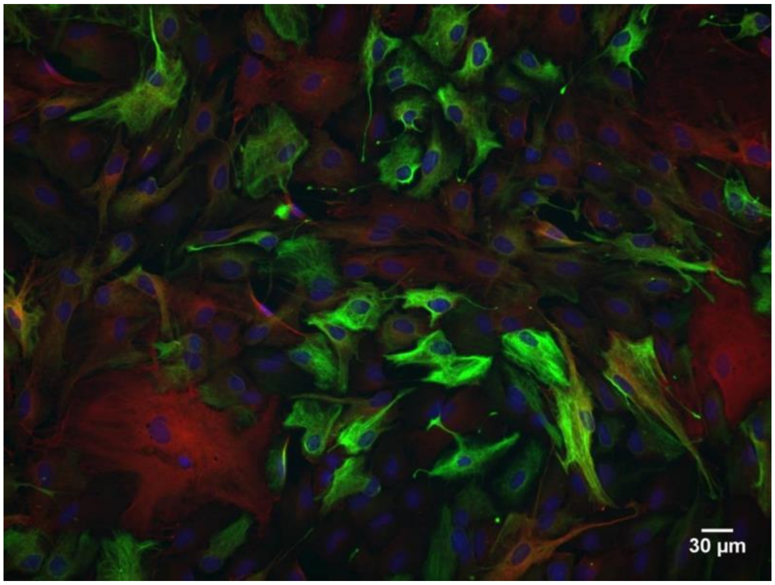

Supplementary Figure 2: Astrocyte cultures stained with nuclear marker DAPI (blue) and immunofluorescent markers for structural proteins beta-III tubulin (red) and GFAP (green), reported to be co-expressed in fetal astrocytes[2], show characteristic circular morphology of nuclei within astrocyte cell bodies.

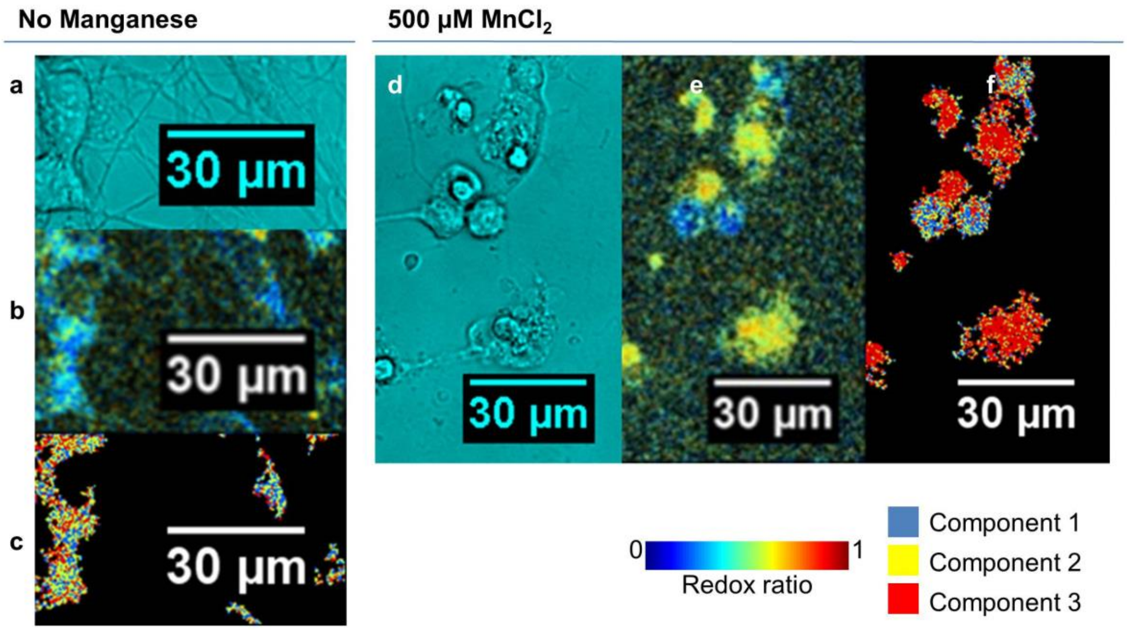

Supplementary Figure 3: Neurons show morphological changes in response to Mn treatment. (a-c) Close-up images of untreated and (d-f) Mn-treated neurons show how morphological shifts including nuclear blebbing and axonal loss (d) colocalize with increases in redox ratio (e) and dominance of component 3 (f). These contrast with the normal morphology (a), lower redox ratio (b), and heterogenous mixture of redox components (c) in untreated cultures.

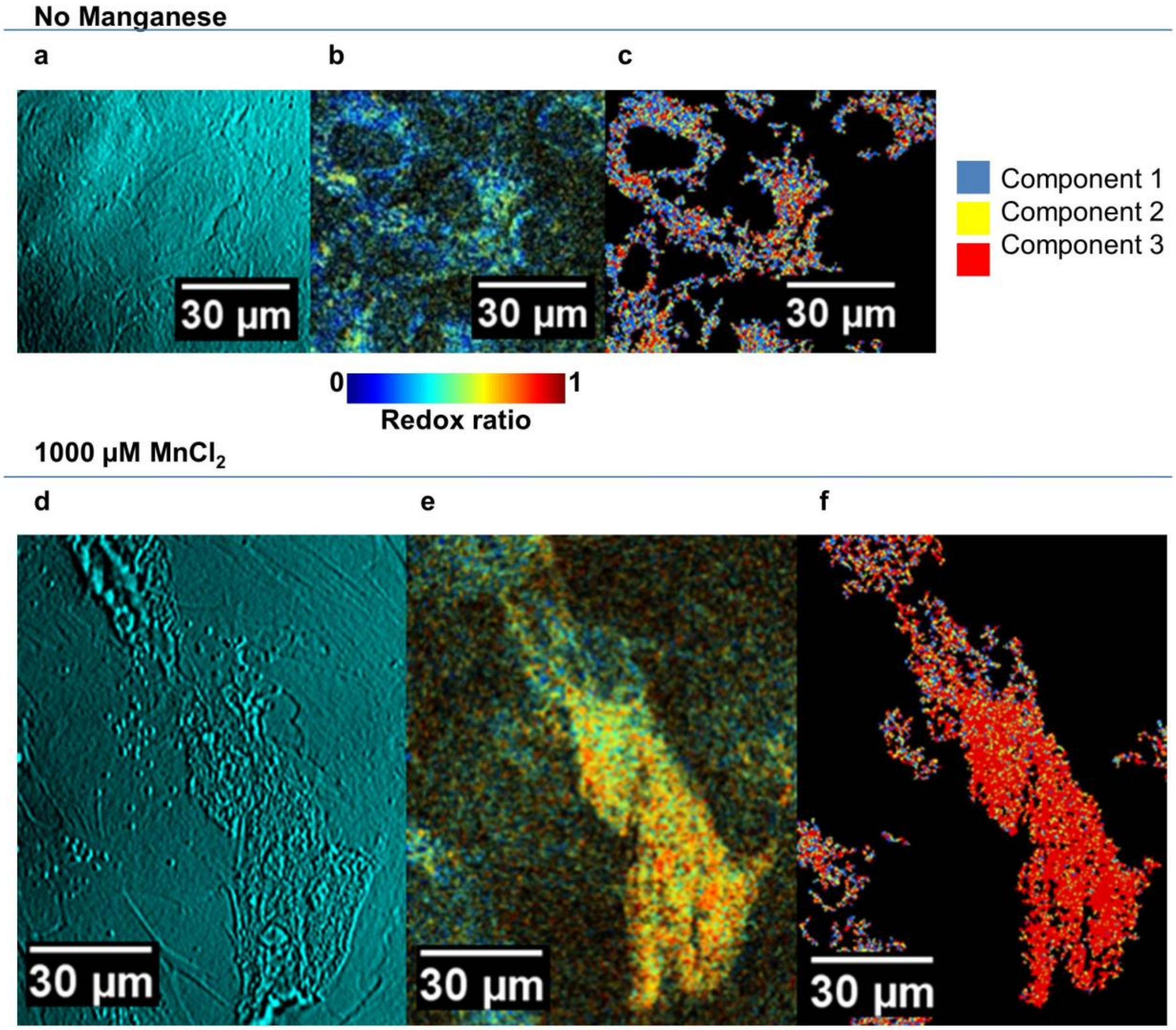

Supplementary Figure 4: Untreated astrocytes show (a) a smooth, confluent morphology, (b) low redox ratio, and (c) a heterogenous mixture of all three redox ratio components. Mn-treated astrocytes exhibit (d) a rougher appearance, (e) increased redox ratio, (f) dominance of component 3

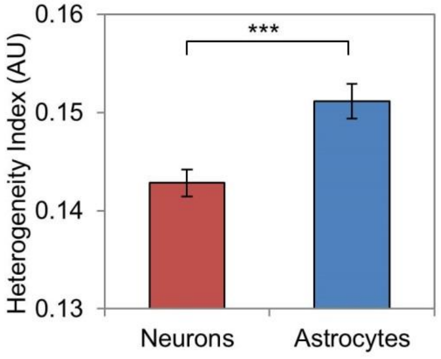

Supplementary Figure 5: Astrocytes show a statistically greater heterogeneity index than neurons. * = p<0.05, **=p<0.01, ***=p<0.001

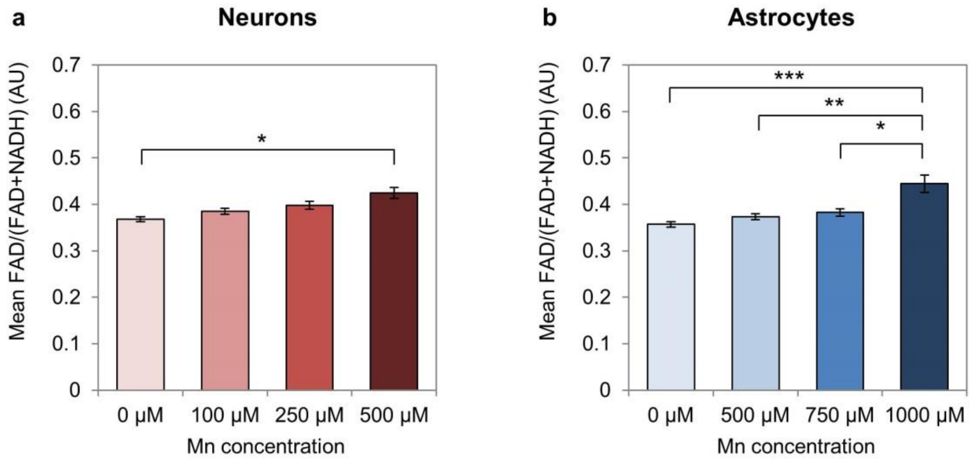

Supplementary Figure 6: Mean optical redox ratios for astrocytes and neurons treated with Mn show less nuanced metabolic shifts than comparisons of redox histogram weight values. (a) Neurons only show a significant increase in redox ratio between 0 and 500μM. (b) Astrocytes show a significant increase in redox ratio at 1000μM relative to the lower dose levels. * = p<0.05, **=p<0.01, ***=p<0.001

1 Shah, A. T., Diggins, K. E., Walsh, A. J., Irish, J. M. & Skala, M. C. In Vivo Autofluorescence Imaging of Tumor Heterogeneity in Response to Treatment. *Neoplasia* **17**, 862-870, doi:10.1016/j.neo.2015.11.006 (2015).

2 Draberova, E. *et al.* Class III beta-tubulin is constitutively coexpressed with glial fibrillary acidic protein and nestin in midgestational human fetal astrocytes: implications for phenotypic identity. *Journal of neuropathology and experimental neurology* **67**, 341-354, doi:10.1097/NEN.0b013e31816a686d (2008).
